# The Path to Discovery of Windup and Central Sensitization

**DOI:** 10.3389/fpain.2022.833104

**Published:** 2022-02-15

**Authors:** Lorne M. Mendell

**Affiliations:** Department of Neurobiology and Behavior, Stony Brook University, Stony Brook, NY, United States

**Keywords:** pain, windup, central sensitization, NMDA receptor, C-fibers, neurokinin receptor

## Introduction

A major advance in our understanding of pain mechanisms emerged from the finding that transmission at spinal synapses in the nociceptive pathway is not fixed but rather exhibits activity-dependence. The first example of this was Windup, initially described in the mid-1960s, showing that successive volleys in peripheral C-fibers evoked bursts of increasing length in spinal neurons. About 15 years later it was found that spinal neurons themselves become sensitized after painful stimuli. These findings prompted many advances in our understanding of how the input from nociceptors is processed and how it might be manipulated to reduce clinical pain. Despite their differences, these two processes share mechanisms, particularly the afferent fibers responsible for activating them and the neurotransmitter/receptor systems involved. The purpose of this brief review is to examine the path leading to their discovery and later to elucidation of the responsible mechanisms.

## Early Experiments Leading to Windup

The discovery of Windup emerged from earlier work on the afferent fibers whose stimulation evoked pain. Observations in the early 1900s by Erlanger and Gasser (1) documented the variation in fiber diameter in sensory nerves (1) and it was subsequently shown by Bishop and Heinbecker (2) that electrical threshold and conduction velocity of axons is correlated with axon diameter (2). Because intense electrical stimulation of peripheral nerves activating the smallest peripheral axons, especially the unmyelinated C-fibers, was required to elicit pain (3), it was expected that individual C-fibers would be activated exclusively by potentially nociceptive stimuli. This was not strongly supported in early electrophysiological studies which challenged the idea that pain was the result of activity in a labeled line activated exclusively by intense stimuli (4). This prompted efforts to see whether the central actions of C-fibers might better explain their relationship to pain. It should be noted, however, that later experiments by Burgess and Perl in animals (5) and by LaMotte and colleagues in humans (6) and others provided clear evidence for afferent fibers activated specifically by intense, potentially damaging stimuli.

The initial approach to determine possible unique central effects of C-fibers was to compare the *presynaptic* effects produced in neighboring sensory fibers by volleys in large myelinated A-fibers and small unmyelinated C-fibers (7). Volleys in large fibers evoked presynaptic inhibition of synaptic input from neighboring afferent fibers whereas volleys in small fibers caused presynaptic facilitation. This originated the concept of a balance between small and large fiber inputs on the level of transmission into the spinal cord which was elaborated by Melzack and Wall (8) into the Gate Theory of Pain [see also (9)].

**Figure 1:**
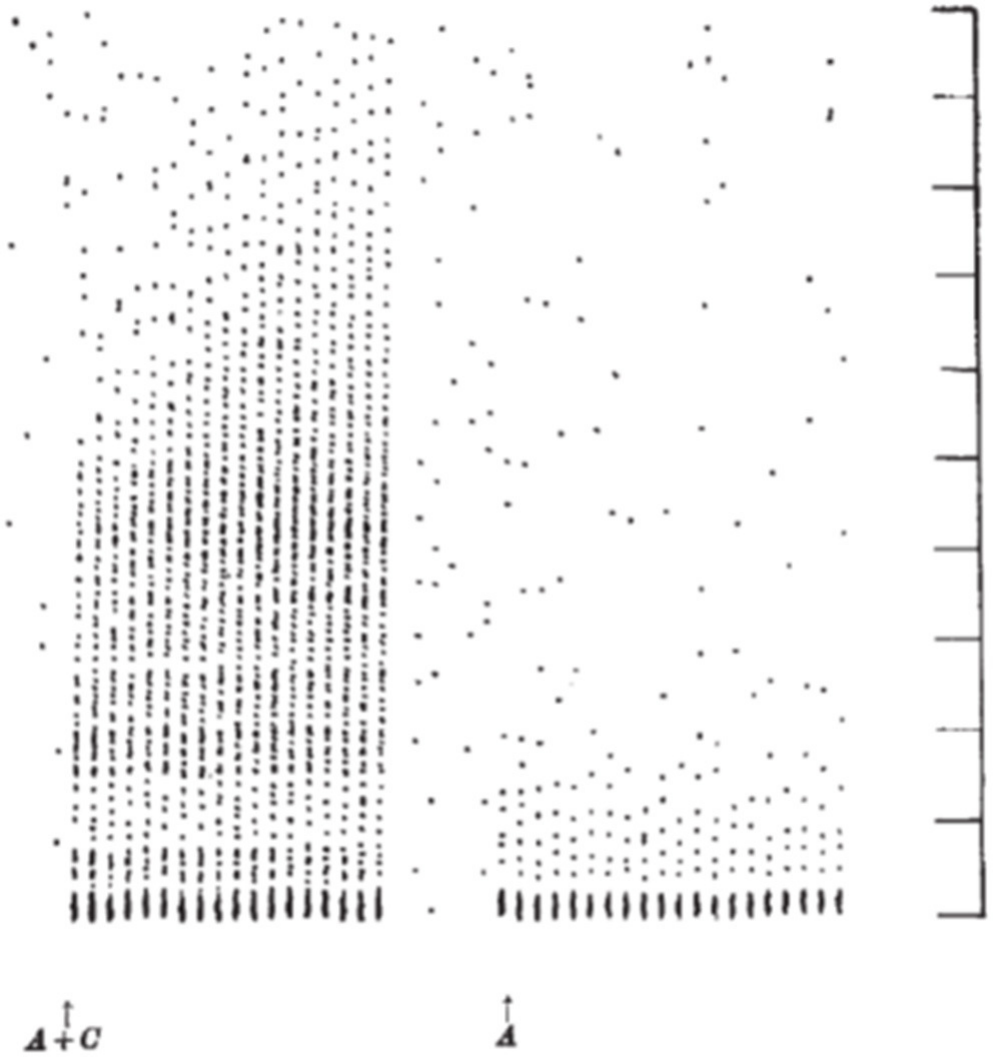
Dot raster display of response of an axon in the dorsolateral lumbar spinal cord in a spinal cat to repetitive stimulation of the ipsilateral sural nerve at a strength to activate A- and C- fibers (left) and A-fibers (right). Each response is shown as a vertical line of dots with each dot representing a single action potential. The stimuli were delivered every 1.3 s. Vertical markers denote 100 ms intervals. The response to the 20 A-fiber stimuli (right) consists of a high frequency burst lasting about 25 ms followed by a silent period and then resumption of spontaneous activity. When C- fibers were also stimulated (left), later activity was also observed. Notably, the late discharge increased in length with repetition of the stimulation (18 delivered in total) (From Figure 2 of Mendell and Wall, Nature, April 3, 1965, with permission).

Mendell and Wall (10) and Mendell (11) extended these results by comparing the *postsynaptic* effects elicited by volleys in peripheral A- and C- fibers. As shown by Wall in previous work (12), stimulating large diameter afferents led to a brief, high frequency repetitive discharge in spinal neurons with a short latency appropriate for the afferent fiber conduction velocity. When the intensity of the electrical stimulus to the peripheral nerve was increased to activate slower conducting C- fibers, a second burst discharge was observed at the expected long latency for the arrival of the C-fiber volley. Unexpectedly, in contrast to the strong inhibition of the activity observed after the A-fiber evoked discharge, the neuron not only exhibited an extended discharge, but this discharge, lengthened with each presentation of the electrical stimulus. Figure 1 demonstrates a spinal neuron discharging at short latency to A-fiber input and at long latency to C-fiber input. The A-fiber response does not change with successive stimuli whereas the C-fiber response increases in duration. This lengthening discharge was called Windup because successive C- fiber volleys were said to be “winding up” the spinal neuron's activity. Neurons often continued to discharge for several seconds after cessation of the stimulation [(10); see also (13) and (14)]. Notably, the minimum stimulation rate to elicit windup in these experiments was about 0.33 Hz implying that each stimulus evoked facilitation lasting about 3 s. This facilitation was synaptic and not hormonal or due to generalized changes (e.g., blood pressure increase) in the CNS since it was unilateral.

The contribution of the C-fiber input to pain was confirmed in spinal neurons by finding that those with C-fiber input exhibiting windup responded to gentle, non-nociceptive brushing of the receptive field (mediated by the peripheral A-fibers) and increased their firing substantially when the skin was pinched to deliver a nociceptive stimulus (mediated by peripheral C-fiber nociceptors). They were called wide dynamic range (WDR) neurons (11) because they responded differentially over a range of stimulus intensities. Neurons without C-fiber input also responded vigorously to brushing of the receptive field but did not respond at a higher level when the skin was pinched. Subsequent studies demonstrated other neurons in the superficial dorsal horn that discharge only in response to high intensity stimuli. Nociceptive specific and WDR neurons became identified as the major classes of neurons responding to nociceptive stimuli (15).

Windup was considered to be intimately associated with pain initially because it was generated by volleys in C-fibers. Importantly, these observations were in accord with clinical studies of Collins and Nulsen (16) that patients report more intense pain in response to repetitive stimulation of C-fibers at frequencies similar to those producing windup of neurons in the spinal dorsal horn, even when volleys in large peripheral A-fibers were prevented from reaching the spinal cord by application of cold block to the peripheral nerve central to the stimulating electrodes.

## Mechanisms of Windup

The mechanism of windup was not apparent at the time of its discovery. Coming soon after the discovery of the opposite presynaptic effects of A- and C- fibers (7), Mendell and Wall (10) suggested that the inhibition and facilitation of burst activity might be a consequence of A-fiber input inhibiting all input to postsynaptic neurons immediately after the burst that they evoked, whereas C-fiber input enhanced subsequent inputs, but this received no experimental support (9). At the time reverberating circuits involving dorsal horn neurons were suggested as a possible mechanism; evidence for this has recently been proposed (17). Temporal summation of synaptic potentials was not considered as a likely mechanism because those that had been recorded lasted of the order of 10 s of milliseconds, not seconds. However, several years later the response of neurons in the ventral horn to C-fiber stimulation was shown to undergo windup and intracellular recordings from these neurons revealed synaptic potentials lasting seconds, long enough for the temporal summation required to obtain windup (18). Since the small afferent fibers responsible for these long lasting synaptic effects do not project directly to motor neurons but rather to neurons in the superficial dorsal horn (19), it was assumed that these very slow synaptic effects would be initiated there and transmitted ventrally. Slow excitatory postsynaptic potentials (EPSPs) in lamina II neurons were demonstrated in subsequent studies (20).

The discovery of the transmitters and receptors mediating these long lasting EPSPs was important in understanding the mechanisms of windup. The identification of a well-defined population of small diameter afferent fibers innervating the skin that responded selectively to high intensity stimuli, either mechanical, thermal or both (5) was a crucial link in the elucidating the process. The somata of these sensory neurons, called nociceptors, were found to express peptides such as substance P and CGRP (21) whose release activated neurokinin receptors on spinal dorsal horn neurons and evoked depolarization lasting of the order of seconds (22). Blockade of the neurokinin receptors reduced windup confirming a role for these peptides in its generation (23). An additional crucial finding was the discovery of a second excitatory glutamate receptor, the NMDA receptor (24), which contributed to slow depolarization. This receptor, prominent in the superficial dorsal horn where C-fibers terminate, was shown to be subject to Mg^2+^ block at normal resting potentials. The temporal summation of the long lasting synaptic depolarization was suggested to relieve the Mg^2+^ block of the NMDA receptors in a cumulative manner with successive stimuli thus increasing postsynaptic depolarization and spike number required for windup. The role of NMDA receptors in windup was confirmed by the ability of NMDA antagonists to block windup without interfering significantly with the initial response to C-fiber volleys (25). Thus, today the unique peptide transmitter content of nociceptors and the NMDA component of their glutamate receptors in the dorsal horn are believed to be major contributors to the synaptic effects of C-fibers in the dorsal horn and thus to windup (26).

## Central Sensitization

The discovery of windup was significant in demonstrating plasticity of the response of dorsal horn neurons to nociceptive stimuli. But interpretation of its role in the pain phenotype was limited because it was produced unphysiologically by electrically initiated nerve volleys in C-fibers. The possibility that pain following injury is secondary to a tissue injury was explored by Woolf (27) who examined changes in the flexor reflex after thermal injury to the skin. Motor neurons were used as a proxy for neurons in the superficial dorsal horn because their position in the final common path receiving inputs from a population of interneurons made their responses more stable and reliable. He reported a gradual increase lasting an hour or more in spike discharge of the motor neurons from stimulating the injured region and even to stimulation of the paw outside the injured area, a change known as secondary hyperalgesia. This expansion of input to flexor motor neurons extended even to skin contralateral to the injured paw. These findings were suggestive of changes in excitability beyond the injured afferent fibers.

The injury also gradually enhanced the windup of flexor motor neurons to sural nerve stimulation, particularly from C-fibers and A-delta fibers. Remarkably, completely anesthetizing the injured foot locally did not prevent or abolish this added response from sural C and A-delta fibers suggesting that the sensitization had a central origin, i.e., central sensitization. Woolf's conclusion that “pain hypersensitivity following injury may be due to changes within the central nervous system as well as at the site of the injury” continues as a dominant theme in the study of pain mechanisms (28). Significantly, Lamotte et al. and Raja et al. have demonstrated a comparable central sensitization in humans (29, 30).

An important advance came from the finding that the late, long lasting component of the response of dorsal horn neurons induced by pain- producing formalin injected into the paw could be blocked by NMDA receptor antagonists with no effect on the initial, brief response (31). This led to the conclusion that central sensitization, like windup, requires activity in NMDA receptors (32). It is also facilitated by activation of neurokinin receptors causing slow depolarization which relieves the Mg^2+^ block of NMDA receptors in the dorsal horn interneurons. Unlike windup, central sensitization can also be caused by agents such as BDNF and NO released into the dorsal horn from nociceptors as a result of peripheral inflammation or nerve injury [reviewed in (33)]. The common element in these dorsal horn mechanisms reflects the entry of Ca^2+^ into the dorsal horn neurons that activates multiple second messengers such as CaMKII and PKC leading to increased excitability. This underlies recruitment of the subliminal fringe of second order neurons to increase receptive field size, etc. These central changes also allow previously ineffective inputs such as large diameter A-fibers to activate these neurons in the nociceptive pathway leading to painful sequelae such as allodynia.

## Comparing Windup and Central Sensitization

Although windup and central sensitization bear certain similarities, notably their activation by C-fibers and their dependence on activity in NMDA and neurokinin receptors, they are not equivalent [see extensive discussion in (34)]. Windup is homosynaptic and requires synchronous volleys in small diameter afferents that produce long lasting synaptic potentials. These summate to more and more depolarized levels with increasing numbers of stimuli until a maximum response is reached, usually in less than a minute. Activation of NMDA and neurokinin receptors in dorsal horn neurons by these C-fiber volleys enhances the effects of the central summation, particularly the length of the impulse discharge (25). Central sensitization also features activation of neurokinin and NMDA receptors increasing the excitability of the postsynaptic neuron. However, volleys in C-fibers and temporal summation of their synaptic responses are not required, only a diffuse barrage in nociceptors leading to a gradual activation of NMDA and neurokinin receptors and increased responsiveness of central neurons, even to unstimulated afferents, i.e., heterosynaptic facilitation. This builds over many minutes, perhaps hours and decays much more slowly than after windup.

Windup does result in the discharge of spinal neurons whose activity persists after cessation of the stimulation (10), but the duration of this activity is briefer than after central sensitization. The original windup papers did not use the term sensitization. However, like central sensitization, windup triggers expansion of the receptive field of activated wide dynamic range spinal neurons (35), but it is not required for central sensitization to occur (36).

## Discussion

Both windup and central sensitization gradually enhance output from spinal neurons in the nociceptive pathway as input persists until a maximum discharge is reached. This elevated discharge can cause painful conditions. For example the increasing levels of spinal neuron discharge with repeated nerve volleys in C-fibers (i.e., the windup) correlates with increased levels of pain in human subjects during such stimulation (16). Similarly, increased levels of pain from skin outside a region of skin sensitized by capsaicin has been reported in human subjects after localized capsaicin application. This secondary hyperalgesia results from central sensitization because there is no change in nociceptor discharge from the sensitized skin outside the capsaicin treated region (29). Thus both windup and central sensitization are models for pain in humans. The basic work on windup and central sensitization has revealed clear differences in the protocols for evoking them but important similarities in the afferent fibers and neurotransmitters responsible for them. Since both mechanisms occur in humans, they remain significant areas for future investigation.

## Author Contributions

The author confirms being the sole contributor of this work and has approved it for publication.

## Conflict of Interest

The author declares that the research was conducted in the absence of any commercial or financial relationships that could be construed as a potential conflict of interest.

## Publisher's Note

All claims expressed in this article are solely those of the authors and do not necessarily represent those of their affiliated organizations, or those of the publisher, the editors and the reviewers. Any product that may be evaluated in this article, or claim that may be made by its manufacturer, is not guaranteed or endorsed by the publisher.
